# Matrix metalloproteinases are hallmark early biomarkers and therapeutic targets in FSHD

**DOI:** 10.1172/jci.insight.195104

**Published:** 2025-09-18

**Authors:** Usuk Jung, Erdong Wei, Haseeb Ahsan, Ana Mitanoska, Kenric Chen, Michael Kyba, Darko Bosnakovski

**Affiliations:** Department of Pediatrics and Lillehei Heart Institute, University of Minnesota, Minneapolis, Minnesota, USA.

**Keywords:** Cell biology, Muscle biology, Fibrosis

## Abstract

Matrix remodeling by metalloproteinases (MMPs) is essential for maintaining muscle homeostasis; however, their dysregulation can drive degenerative processes. By interrogating biopsy RNA-Seq data, we showed that MMP expression correlated with disease severity in facioscapulohumeral muscular dystrophy (FSHD). In the *iDUX4pA* FSHD mouse model, MMP levels also progressively increased in response to double homeobox 4–induced (DUX4-induced) muscle degeneration. Single-cell RNA-Seq further identified fibroadipogenic progenitors (FAPs) and macrophages as the primary sources of MMPs, particularly MMP2, MMP14, and MMP19, in dystrophic muscle. Treatment with the pan-MMP inhibitor batimastat alleviated inflammation and fibrosis, improved muscle structure, and decreased the number of FAPs and infiltrating macrophages. These findings underscore the role of MMPs in driving muscle degeneration in FSHD, highlight MMPs as functional biomarkers of disease, and support MMP inhibitors as a DUX4-independent therapeutic approach to limit fibroadipogenesis and promote muscle regeneration.

## Abstract 2

Matrix metalloproteinases (MMPs) are early and progressive drivers of FSHD muscle degeneration, and their inhibition alleviates pathology, offering a DUX4-independent therapeutic strategy.

## Introduction

Facioscapulohumeral muscular dystrophy (FSHD) is a prevalent genetic muscle disorder characterized by progressive weakening and atrophy of skeletal muscles in the face, trunk, and limbs (1, 2). This debilitating condition arises from incomplete silencing of the D4Z4 repeat array and aberrant expression of the double homeobox 4 (*DUX4*) gene (3–5). Under normal conditions, DUX4 expression is restricted to zygotic genome activation (6–9); however, its misexpression has deleterious consequences, including cell death (10, 11) and impaired differentiation (11, 12). In skeletal muscle, this leads to inflammation, excessive extracellular matrix (ECM) deposition, compromised microvasculature, and impaired regeneration (13–16). In the *iDUX4pA* (*iDUX4*) mouse model, transient DUX4 expression in myofibers leads to long-term expansion of fibroadipogenic progenitors (FAPs) (17), the cell type responsible for fibrosis and fatty infiltration of muscle tissue (18). A similar expansion of FAPs has now been observed in human FSHD biopsies (19, 20). In addition to increasing in number, FAPs undergo a phenotypic shift from a pro-regenerative to a pro-fibrotic state (21–23). This transition is mediated by a range of cytokines, including TGFB, bone morphogenetic protein 4 (BMP4), TNFA, IL-4, and IL-13. It is further facilitated by matrix metalloproteinases (MMPs), which are thought to be primarily secreted by infiltrating mononuclear cells (24–26).

MMPs play critical roles in regulating the ECM under both physiological and pathological conditions (27, 28). These enzymes degrade key ECM components such as collagen and laminin, releasing growth factors that influence cellular activation, migration, inflammation, angiogenesis, and tissue regeneration (29–31). Dysregulated MMP activity has been implicated in muscular dystrophies, where it disrupts the ECM–cytoskeleton network, leading to sarcolemmal damage, myofiber necrosis, and impaired muscle regeneration (32, 33). In the *mdx* mouse model of Duchenne muscular dystrophy (DMD), MMP9 inhibition has been shown to reduce fibrosis, promote myofiber regeneration, and improve muscle structure and function (32–34). Although the role of MMPs in FSHD pathology has not been investigated, elevated MMP levels are observed in RNA-Seq data from the *iDUX4* FSHD mouse model (15, 16), suggesting a potential contribution to disease progression.

In this study, we evaluated MMP expression in muscle biopsies from patients with FSHD and found a strong correlation with disease severity. Notably, elevated MMP levels were also detected in short TI inversion recovery–negative (STIR^–^), clinically uninflamed muscles, suggesting a potential role for MMPs in the early stages of FSHD pathogenesis. Using the *iDUX4* mouse model, we observed a similar pattern of MMP induction correlated with DUX4-induced muscle damage. We investigate the dystrophic muscle by single-cell RNA-Seq (scRNA-Seq) to understand which cell types are the primary sources of MMP production in DUX4-affected muscles and test pharmacological inhibition of MMPs using the pan-MMP inhibitor batimastat (BB-94). Our findings reveal a critical role for MMPs in FSHD pathology and highlight MMP inhibition as a promising strategy to modulate inflammation and fibrosis and to promote muscle regeneration.

## Results

### MMP gene expression is elevated in FSHD muscle and correlates with disease severity.

**Figure 1:**
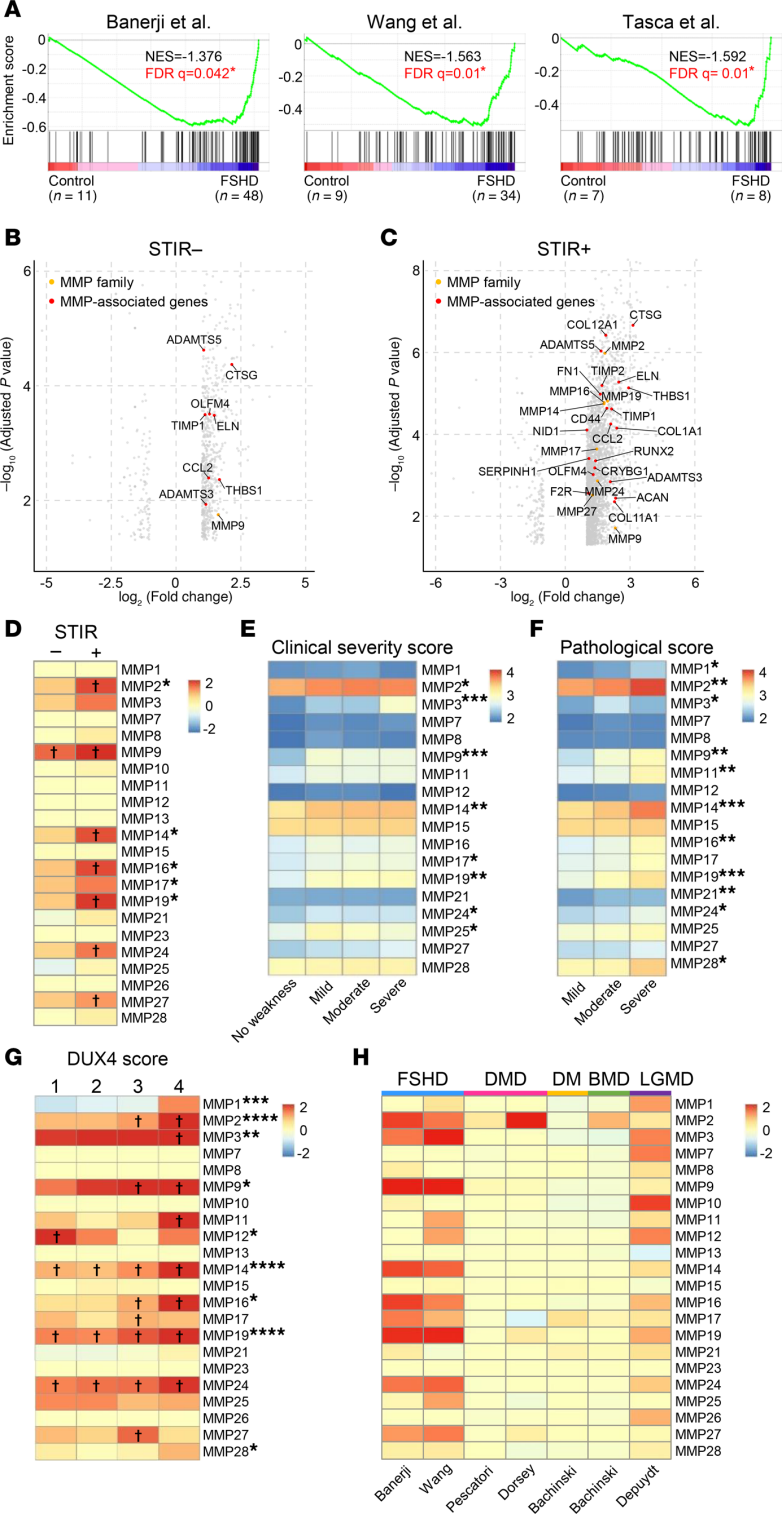
Elevated MMP and MMP-associated genes in FSHD patient biopsies. (**A**) GSEA shows enrichment of 23 MMPs and 79 MMP-associated genes in biopsy samples from healthy donors (controls) and patients with FSHD across 3 independent studies (38–40). Asterisks indicate significant enrichment (FDR < 0.25). (**B** and **C**) Volcano plots display differential expression MMP family and MMP-associated genes in STIR^−^ (uninflamed) (**B**) and STIR^+^ (inflamed) (**C**) FSHD biopsies compared with healthy controls from the Banerji dataset. (**D**) Heatmaps illustrating MMP gene expression patterns in STIR^−^ and STIR^+^ samples from the Banerji dataset. Cross marks indicate statistical significance compared with control. ^†^*P* < 0.05 by Student’s 2-tailed *t* test. Asterisks indicate significant differences between STIR^−^ and STIR^+^. *P < 0.05 by Student’s 2-tailed *t* test. (**E** and **F**) Heatmaps showing the correlation between clinical severity score (**E**) and pathological score (**F**) and MMP expression in initial visit biopsy samples from the Wang dataset. Asterisks indicate statistical significance. *P < 0.05, **P < 0.01, and ***P < 0.001 by 1-way ANOVA. (**G**) Heatmaps depicting the increased MMP levels in line with the expression of DUX4-associated biomarker genes in Wang dataset. Cross marks indicate statistical significance compared with control. ^†^*P* < 0.05 by Student’s 2-tailed *t* test. Asterisks indicate significant differences across DUX4 score groups 1, 2, 3, and 4. *P < 0.05, **P < 0.01, ***P < 0.001, and ****P < 0.0001 by 1-way ANOVA. (**H**) Summary of MMP expression across various muscular dystrophies. Expression in each disease cohort was compared with the corresponding healthy control within each study.

To investigate the role of MMPs in FSHD pathogenesis, we analyzed the expression of 23 MMP family genes (35–37) and 79 MMP-associated genes (STRING database v12.0) across 3 independent RNA-Seq datasets comprising muscle biopsies from a total of 90 patients with FSHD (38–40) (Supplemental Table 1). Gene set enrichment analysis (GSEA) revealed significant enrichment of both MMP family genes and MMP-associated genes in FSHD muscle samples compared with controls (Figure 1A).

We next evaluated whether MMP expression correlates with muscle disease state. The studies by Banerji et al. and Wang et al. employed MRI-guided biopsies and sufficiently sized cohorts, enabling a clear distinction between affected inflamed (STIR^+^) and nonaffected (STIR^−^) muscle tissues (39, 40). Comparative analyses revealed significantly higher expression of MMP and MMP-associated genes in STIR^−/+^ compared with control biopsies (Figure 1, B and C, and Supplemental Figure 1A). In the Banerji dataset, *MMP9* was the only MMP significantly upregulated in STIR^−^ biopsies relative to controls, whereas 7 out of 23 MMPs were significantly upregulated in STIR^+^ biopsies (Figure 1, B−D, and Supplemental Figure 1B). A similar trend was evident among MMP-associated genes, with 8 genes upregulated in STIR^−^ and 20 genes in STIR^+^ biopsies (Figure 1, B and C). The Wang dataset revealed a comparable expression pattern, with *MMP19* and *MMP24* upregulated in STIR^−^ samples and 9 additional MMPs elevated in STIR^+^ biopsies (Supplemental Figure 1, A and C). Similarly, 15 MMP-associated genes were upregulated in STIR^−^ and 25 in STIR^+^ muscles (Supplemental Figure 1A). Cross-study comparison identified *MMP2*, *-9*, *-14*, *-16*, *-19*, *-24*, and *-27* as consistently upregulated in STIR^+^ muscle across both datasets (Figure 1C and Supplemental Figure 1A).

To further validate our findings, we analyzed data from Wong et al., a longitudinal study in which muscle biopsies were collected from the same muscle group in patients with FSHD at baseline and at a 1-year follow-up (41). Transcriptomic profiles were correlated with clinical severity scores, classifying patients as having no weakness or mild, moderate, or severe symptoms, and with histopathological scores categorizing pathology as mild, moderate, or severe (41). In the initial visit, 8 MMPs showed significant correlations with clinical severity, whereas 9 MMPs did so at the follow-up visit (Figure 1E and Supplemental Figure 1D). Notably, MMP expression levels were even more strongly associated with pathological severity, with nearly half of the MMPs significantly correlated at the initial visit and one-third remaining significantly associated at follow-up (Figure 1F and Supplemental Figure 1E).

Finally, we analyzed data from Wang et al., dividing the cohort into 4 groups based on expression levels of DUX4-related biomarker genes (39). Strikingly, 9 out of 22 MMPs, including *MMP2*, *-3*, *-9*, *-14*, and *-24*, were significantly upregulated in correlation with DUX4 biomarker expression, suggesting a potential link between DUX4 activity and MMP induction in muscle tissue (Figure 1G).

To determine whether MMP induction is a general feature of muscle degeneration or specific to FSHD, we compared the expression of MMP and MMP-associated genes in FSHD biopsies (39, 40) with those from patients with DMD, myotonic dystrophy (DM), Becker muscular dystrophy (BMD), and limb-girdle muscular dystrophy type 2L (LGMD 2L). Although elevated MMP expression was observed across multiple dystrophies, FSHD samples exhibited the highest and most widespread induction of both MMP and MMP-associated genes (Figure 1H and Supplemental Figure 2). This distinctive expression pattern suggests that broad MMP dysregulation may play a particularly prominent role in the molecular mechanisms underlying FSHD.

Taken together, these results demonstrate that induction of MMP and MMP-associated genes is a hallmark of FSHD muscle, correlates with disease severity, and may serve as an early biomarker even in clinically uninflamed STIR^−^ muscle.

### MMP gene expression correlates with disease progression in the FSHD mouse model.

**Figure 2:**
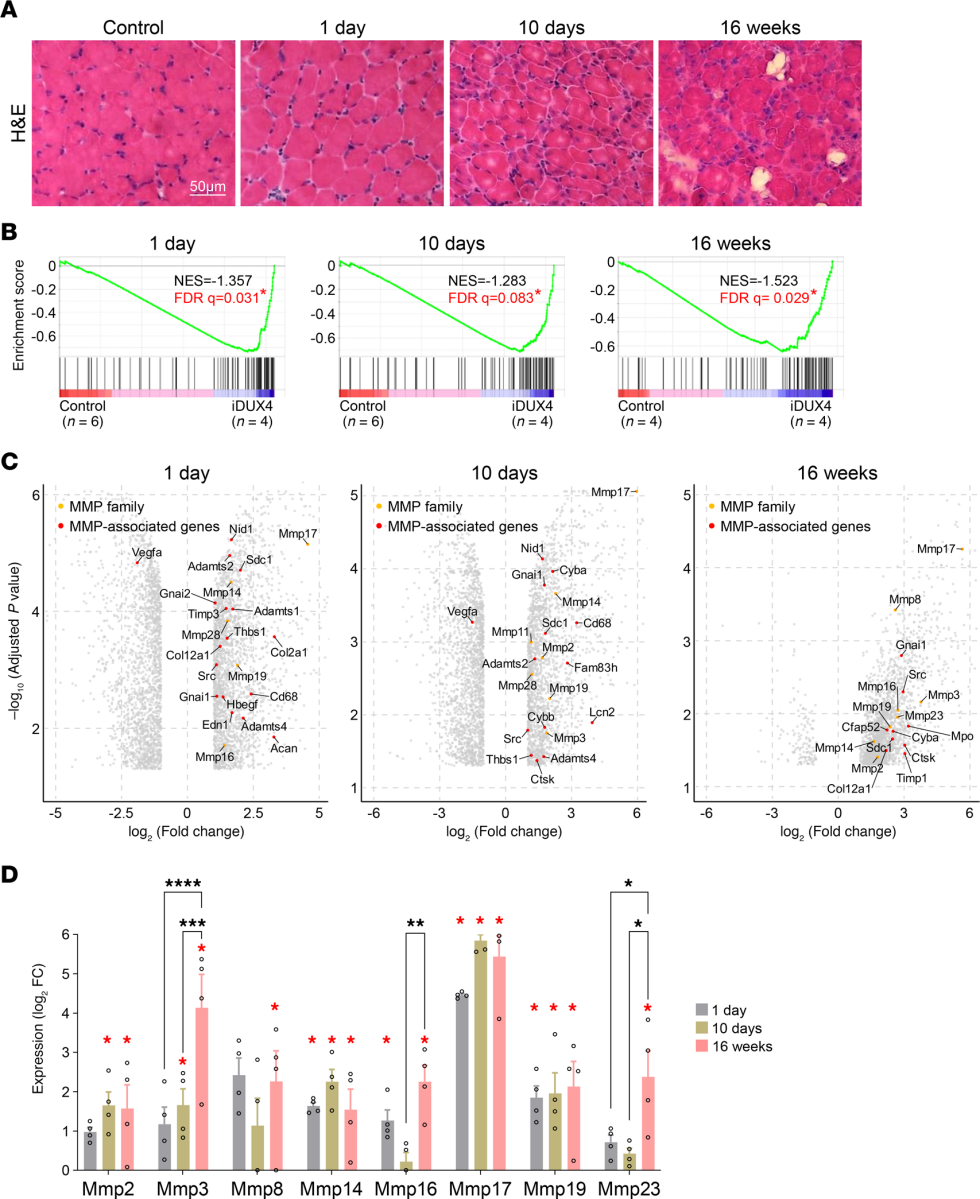
MMP expression correlates with disease stage in *iDUX4* mice. (**A**) Representative H&E-stained images of muscle tissues from uninduced *iDUX4* mice (control) and at 1 day, 10 days, and 16 weeks following DUX4 induction (100 mg/kg/d dox). Scale bar: 50 μm. (**B**) GSEA reveals progressive enrichment of MMPs and MMP-related genes at 1 day, 10 days, and 16 weeks following DUX4 induction, compared with uninduced, age-matched *iDUX4* control mice. Asterisks denote statistically significant enrichment (FDR < 0.25). Each induced group includes 4 mice; control groups consist of 6 mice for the 1- and 10-day time points and 4 mice for the 16-week time point. (**C**) Volcano plots display differential expression of MMP and MMP-associated genes at 1 day, 10 days, and 16 weeks after DUX4 induction, compared with control uninduced *iDUX4* mice. (**D**) Bar graph showing fold changes in MMP gene expression at various time points following DUX4 induction, normalized to age-matched, uninduced *iDUX4* mice. Data are presented as mean ± SEM. Red asterisks indicate statistically significant differences between each DUX4-induced group and its respective time-matched control (*P < 0.05, Student’s 2-tailed *t* test). Black asterisks indicate significant differences among DUX4-induced time points (*P < 0.05, **P < 0.01, ***P < 0.001, ****P < 0.0001; 1-way ANOVA with Tukey’s post hoc test).

We and others have demonstrated that mouse models with muscle-specific DUX4 expression closely recapitulate the dystrophic pathology observed in patients with FSHD (13–16, 42). To assess whether MMP expression correlates with disease severity in this model, we induced DUX4 expression in skeletal muscle (via HSA-rtTA) using doxycycline (dox; 100 mg/kg/d) and performed transcriptional profiling of the gastrocnemius muscle at 3 key time points: day 1 (early onset), day 10 (acute injury), and 16 weeks (chronic stage) (Figure 2A). Age-matched, uninduced *iDUX4* mice served as controls. We first examined the enrichment of 23 MMP genes and 58 MMP-associated genes (Supplemental Table 2) across these 3 time points using GSEA. Notably, both MMP and MMP-associated gene sets were significantly enriched at all stages, including as early as 24 hours postinduction (Figure 2B), suggesting that MMP activation begins at disease onset and persists throughout chronic DUX4-induced muscle degeneration. Closer analysis revealed early upregulation of *Mmp14*, *-16*, *-17*, -*19*, and -*28* at 24 hours postinduction, with sustained expression through the acute and chronic phases (Figure 2, C and D). With prolonged DUX4 expression, additional MMPs, including *Mmp2*, -*3*, *-8*, and *-23*, were upregulated, implicating them in progressive degenerative processes (Figure 2, C and D). By 16 weeks, 8 MMPs were significantly upregulated in DUX4-affected muscle, reflecting the expression patterns observed in FSHD patient biopsies (Figure 1D and Figure 2D). Induction of MMP2 and MMP14 was further confirmed by immunofluorescence (Supplemental Figure 3).

These results indicate that MMP and MMP-associated gene expression in the *iDUX4* model is strongly linked to disease progression and severity, further validating this model for investigating MMP-related mechanisms in FSHD.

### Cell-specific MMP expression in DUX4-affected muscles.

**Figure 3:**
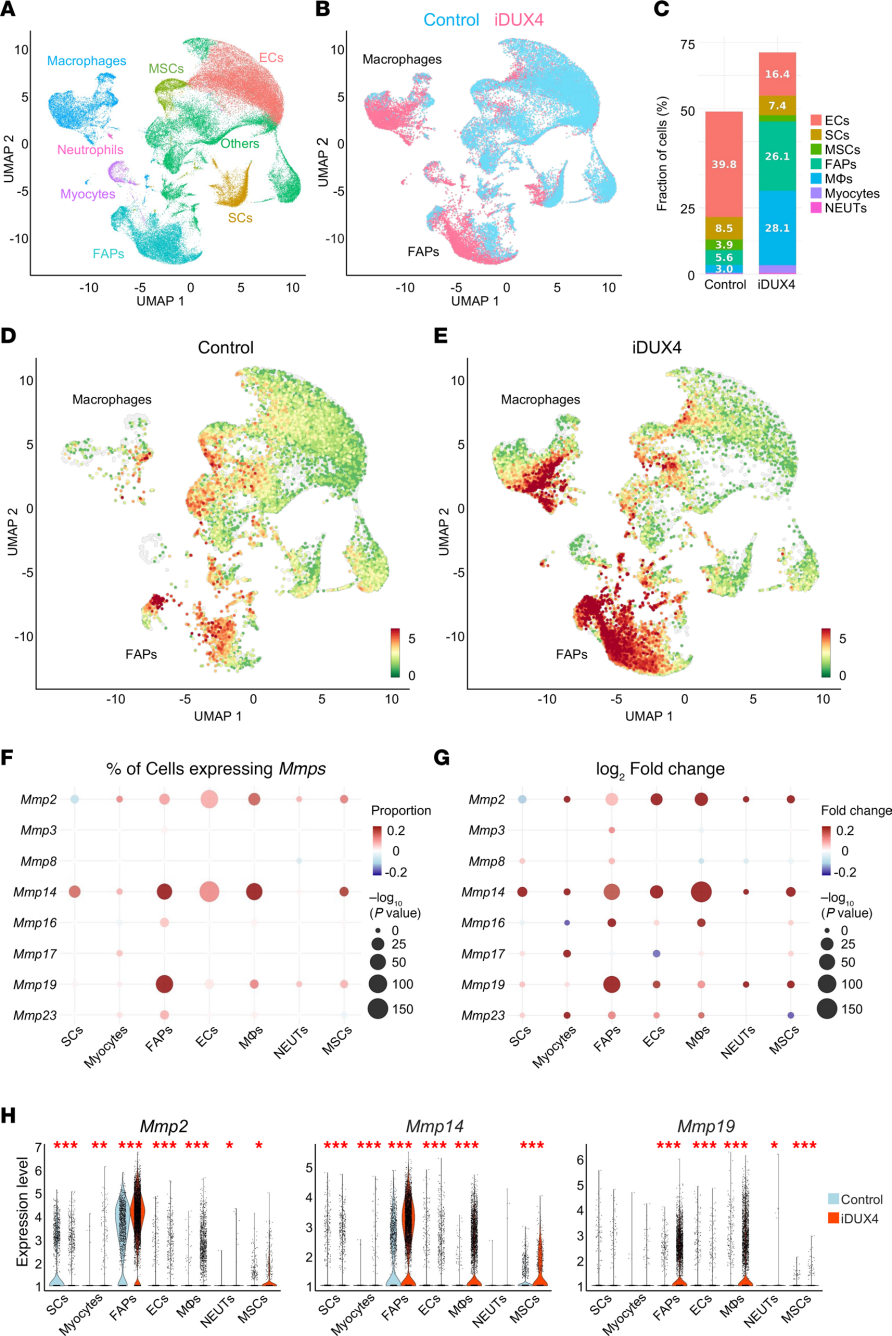
Single-cell resolution of MMP expression in DUX4-affected skeletal muscle. (**A**) UMAP plot of merged single-cell data from uninduced WT (control) and dox-induced iDUX4 mice (10 days on 625 mg/kg dox chow), with clusters annotated by cell types. (**B**) UMAP comparison of cell clusters between control and DUX4-induced muscles. (**C**) Proportional representation of major cell populations in control and DUX4-induced muscles. (**D** and **E**) UMAP plots showing the spatial distribution and cellular origin of MMP expression in control (**D**) and DUX4-induced (**E**) muscles. (**F**) Bubble plots highlighting distinct MMP expression patterns across cell types. (**G**) Normalized fold-change in MMP expression within selected cell populations. (**H**) Violin plots showing expression levels of *Mmp2*, *Mmp14*, and *Mmp19* across annotated cell clusters. Data are presented as mean ± SEM. *P < 0.05, **P < 0.01, ***P < 0.001 by Student’s 2-tailed *t* test. ECs, endothelial cells; MSCs, mesenchymal stem cells; SCs, satellite cells; UMAP, uniform manifold approximation and projection.

To identify the cellular sources of MMP expression in DUX4-affected muscle, we performed scRNA-Seq on pooled skeletal muscles (tibialis anterior [TA], gastrocnemius, and quadriceps) from *iDUX4* mice following 10 days of DUX4 induction via 625 mg/kg dox chow. Age-matched wild-type (*WT*) mice fed the same dox-containing diet served as controls. In total, 40,322 cells were retrieved, 21,897 from controls and 18,425 from DUX4-induced mice. Clustering based on transcriptional signatures identified 25 distinct cell populations (Figure 3A and Supplemental Table 3). Cell types were annotated using canonical markers, including ECs (*Cdh5*), SCs (*Pax7*), myocytes (*Myl1*), MSCs (*Nt5e*), FAPs (*Pdgfra*), macrophages (*CD86*), and neutrophils (*S100a9*) (Supplemental Figure 5 and Supplemental Table 3). DUX4 induction resulted in substantial alterations in cellular composition (Figure 3B), most notably a dramatic expansion of FAPs, from 5.6% in controls to 26.1% in DUX4-induced muscle, and a substantial increase in macrophages, which comprised 28% of the mononuclear cell population (Figure 3C). Additionally, we observed a marked reduction in ECs, a slight decrease in SCs, and an increase in myocytes (Figure 3C).

Combined UMAP analysis was used to visualize the cellular sources of MMP expression. This approach revealed a striking upregulation of MMPs within FAPs and macrophage clusters, identifying these cell populations as the primary contributors to MMP production in DUX4-affected muscle (Figure 3, D and E).

Further analysis revealed distinct patterns of MMP expression across various cell types (Figure 3, F and G). Notably, *Mmp2*, *-14*, and *-19* were broadly and strongly expressed, with the highest levels observed in FAPs and macrophages (Figure 3H). Colocalization of MMP2 and MMP14 in these 2 cell populations was further verified by immunostaining of quadriceps muscle from *iDUX4* mice after 10 days of DUX4 induction (Supplemental Figure 4). While most MMPs were upregulated, a few showed reduced expression in certain cell types, for example, *Mmp2* in SCs, *Mmp8* in neutrophils and MSCs, *Mmp16* in myocytes, *Mmp17* in ECs, and *Mmp23* in MSCs (Figure 3G). Taken together, these findings indicate that during acute *iDUX4*-induced muscle degeneration, MMPs are broadly upregulated across mononuclear cell populations, with FAPs and macrophages serving as the predominant sources.

### MMP inhibition attenuates DUX4-induced muscle damage.

**Figure 4:**
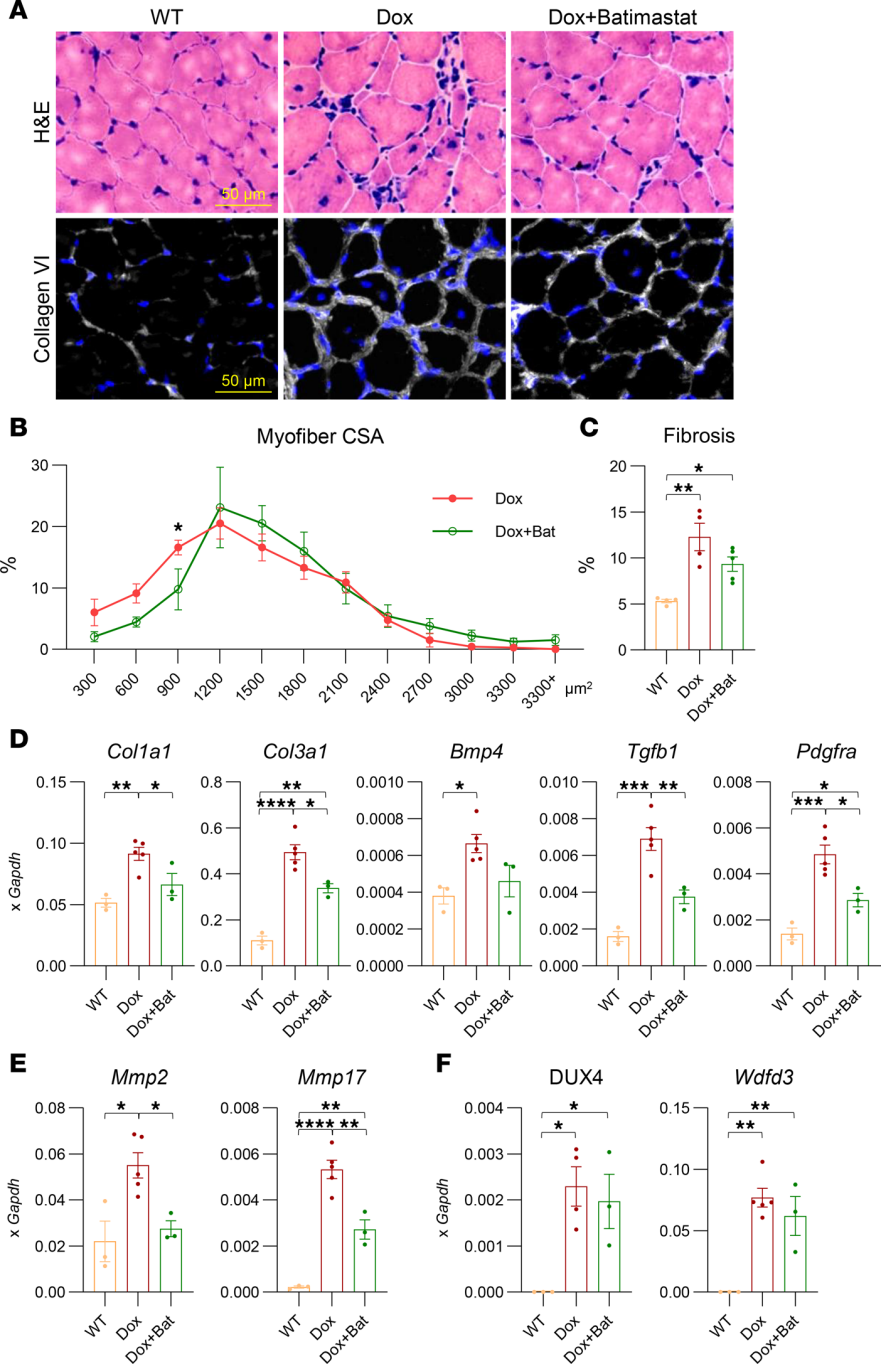
Batimastat treatment improves muscle phenotypes in *iDUX4* mice. (**A**) H&E staining of tibialis anterior (TA) muscle from WT, iDUX4 (Dox), and batimastat-treated iDUX4 mice (Dox+Bat). Immunofluorescence staining of TA muscle for collagen VI (white) and DAPI (blue). Scale bar: 50 μm. All mice were fed dox chow (62.5 mg/kg) for 20 days. Batimastat (2 mg/kg) was administered daily via intraperitoneal injection during the final 10 days of the induction period. (**B**) Distribution of myofiber cross-sectional area (CSA) in iDUX4 mice, with and without batimastat treatment (*n* = 4 per group). Data are shown as mean ± SEM. *P < 0.05 by multiple unpaired *t* tests. (**C**) Quantification of fibrosis based on collagen VI immunostaining in the samples shown in **A**. Data are presented as mean ± SEM. *P < 0.05, **P < 0.01 by 1-way ANOVA followed by Tukey’s post hoc tests (*n* = 4). (**D**) Reverse transcription quantitative PCR (RT-qPCR) analysis of fibrotic markers (*Mmp2*, *Mmp17*, *Col1a1*, *Col3a1*, *Tgfb1*, *Bmp4*, and *Pdgfra*) in gastrocnemius muscle from WT (*n* = 3), Dox (*n* = 5), and Dox+Bat (*n* = 3). Gene expression was normalized to *Gapdh*. (**E**) RT-qPCR analysis of *Mmp2* and *Mmp17*. (**F**) RT-qPCR analysis of *DUX4* and its target gene *Wfdc3*. Data are presented as mean ± SEM. *P < 0.05, **P < 0.01, ***P < 0.001, ****P < 0.0001 by 1-way ANOVA followed by Tukey’s post hoc tests.

To evaluate the functional contribution of MMPs to the dystrophic phenotype in DUX4-affected muscle, we tested the pan-MMP inhibitor batimastat in a model of acute, mild, DUX4-induced FSHD phenotype. Mice were treated with 62.5 mg/kg dox chow for 20 days. Batimastat was administered at a dose of 2 mg/kg by daily i.p. injection, starting on day 10 and continuing through the end of the experiment (Supplemental Figure 6A). Muscle composition was evaluated by H&E staining to assess myofiber structure and mononuclear cell infiltration and by collagen VI immunostaining to visualize ECM deposition (Figure 4A). As previously reported (13), low-dose dox induction (62.5 mg/kg) exhibited a mild dystrophic phenotype characterized by abnormal myofiber size distribution, increased centronucleated myofibers, elevated mononuclear cell infiltration, and interstitial fibrosis compared with *WT* mice (Figure 4A) (13). Batimastat treatment resulted in a moderate but significant improvement in muscle morphology, as indicated by an increase in average myofiber size (Figure 4B). Additionally, collagen VI staining showed a significant reduction in fibrotic ECM deposition in batimastat-treated muscles compared with disease controls (Figure 4, A and C). While fibrosis was reduced and overall muscle histology improved, batimastat did not prevent DUX4-induced muscle mass loss over this relatively short treatment period (Supplemental Figure 6B). Transcriptional analysis verified this improvement, with decreased expression of fibrotic markers (*Col1a1* and *Col3a1*), inflammatory mediators (*Tgfb1*), and ECM remodeling enzymes (*Mmp2* and *Mmp17*) (Figure 4, D and E). Additionally, reduced *Pdgfra* expression suggested decreased FAP abundance or activity (Figure 4D). Treatment had no effect on expression levels of *DUX4* or its downstream target *Wfdc3* (Figure 4F), indicating that the drug does not interfere with the dox-inducible system.

**Figure 5:**
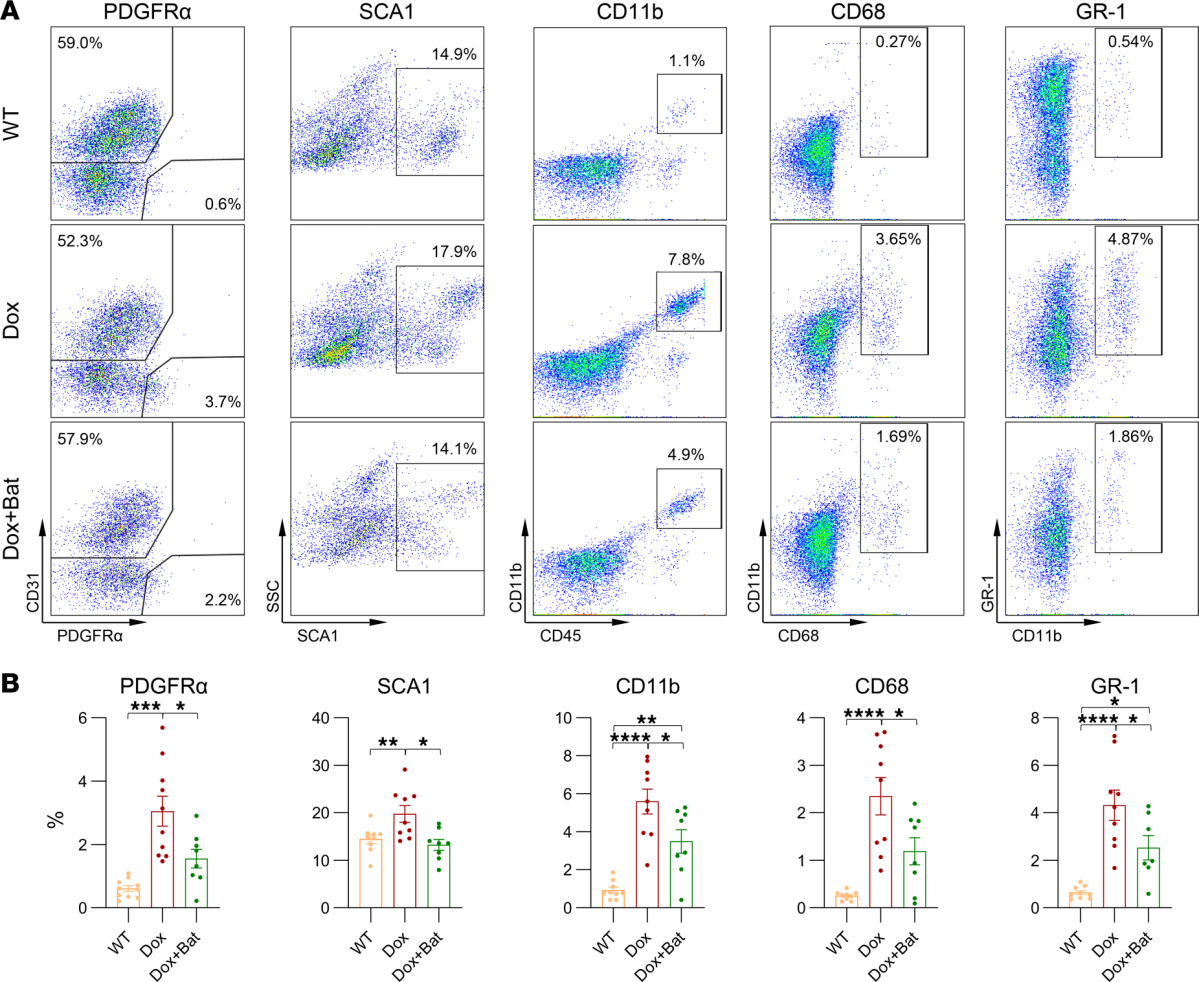
MMP inhibition reduces mononuclear cell infiltration in *iDUX4* mice. (**A**) Representative FACS profile for CD45^–^CD31^–^PDGFRα^+^ (FAPs) or CD31^+^PDGFRα^–^ (ECs), CD45^–^SCA1^+^ cells, CD11b^+^CD68^+^, and CD11b^+^GR-1^+^ (myeloid-derived suppressor cells) in the quadriceps from WT, iDUX4 (Dox), and batimastat-treated iDUX4 (Dox+Bat) mice. (**B**) Quantification of cell populations. Cell frequencies are represented as percentages of the parent cell population. Data are presented as mean ± SEM; *P < 0.05, **P < 0.01, ***P < 0.001, and ****P < 0.0001 by 1-way ANOVA followed by Tukey’s post hoc tests; WT (*n* = 9); Dox (*n* = 9); Dox+Bat (*n* = 8).

FAPs and macrophages, key producers of MMPs, are known to accumulate in dystrophic muscle and play central roles in the *iDUX4* model of FSHD (16). To evaluate the cellular effects of batimastat, we performed FACS analysis on TA muscles. Batimastat treatment significantly reduced the abundance of FAPs, indicated by decreased PDGFRα^+^ and SCA1^+^ cell populations (Figure 5, A and B). To determine whether this reduction was due to direct inhibition of FAP proliferation or an indirect consequence of MMP suppression, we treated FAPs isolated from *WT* and chronically induced *iDUX4* mice with batimastat in vitro. After 24 hours, no significant changes in viability or proliferation were observed, as measured by ATP content and Ki-67 staining, respectively (Supplemental Figure 6, C–E). In addition to reducing FAPs, batimastat significantly reduced inflammatory infiltration in muscle tissue, including both macrophages and broader myeloid cell populations (Figure 5, A and B). Together, these findings demonstrate that batimastat, a pan-MMP inhibitor, effectively improves muscle histopathology in DUX4-induced FSHD by attenuating both fibrotic and inflammatory responses through MMP inhibition.

## Discussion

In this study, we demonstrate that the induction of MMPs is a hallmark feature of FSHD muscle pathology. MMPs, a family of 23 zinc-dependent endopeptidases, are key regulators of ECM remodeling through their ability to degrade a wide range of structural proteins. They are involved in diverse physiological and pathological processes, including tissue repair, inflammation, and fibrosis (43). Dysregulated MMP activity has been implicated in several inflammatory myopathies, neurodegenerative muscular disorders, and muscular dystrophies including DMD and Emery-Dreifuss (27, 32, 33, 44–48). By analyzing transcriptional datasets from 3 independent FSHD studies (38–40), we consistently identified strong correlations between MMP expression, MMP-associated gene networks, and disease severity. Notably, both the expression levels and the number of upregulated MMP and MMP-related genes correlated positively with clinical and pathological severity, as well as with the expression of established FSHD biomarker genes. A particularly intriguing observation was the elevated expression of several MMPs, especially *MMP9*, *-19*, and *-24*, in STIR^–^ FSHD muscle samples, which appear histologically normal and lack signs of active inflammation. This suggests that MMP upregulation may occur early in disease development, prior to overt muscle degeneration, and highlights the potential of MMPs as early functional biomarkers of FSHD progression (49). While the roles of MMP19 and MMP24 remain poorly characterized, the various functions of MMP9 in skeletal muscle homeostasis are well documented. MMP9 expression is transiently induced following a single bout of exercise, suggesting a physiological role in muscle maintenance and remodeling (50, 51). Elevated levels of MMP9 have been reported in patients with DMD compared with healthy controls across multiple studies (52, 53). Similarly, MMP9 induction has been reported in the *mdx* mouse model, where it contributes to fibroadipogenic expansion and muscle degeneration (32, 54). Inhibition of MMP9 activity, using either a nuclear factor-κB inhibitory peptide or MMP9-specific antibodies, has been shown to attenuate fibrosis in both *mdx* and acute muscle injury models (32, 55). Consistent with our findings, a prior study investigating potential serum biomarkers for FSHD in a small cohort of 23 patients and age-matched controls reported elevated MMP9 levels in patient serum (56). Although the difference did not reach statistical significance (*P* = 0.08) and MMP9 was not identified as a reliable serum biomarker in that study, our data demonstrated robust MMP9 upregulation in both patient biopsies and in response to DUX4 expression in muscle fibers. These findings suggest that MMP levels in muscle tissue, rather than serum, may serve as a useful functional biomarker for clinical studies evaluating therapies targeting DUX4. Nevertheless, it will be important to investigate MMP levels at the protein level in FSHD biopsy slides and specimens. Currently available proteomic datasets use mass spectrometry (57, 58), which have discovered differences in high-expressed proteins but are most likely underpowered to detect changes in low-expressed proteins given the regional and patient-to-patient variability intrinsic to FSHD.

In STIR^+^ FSHD muscle, more than one-third of MMPs and related genes were upregulated (Figure 1), highlighting the involvement of proteolytic pathways in FSHD. The most highly expressed MMPs belonged to gelatinase (*MMP2*, *-9*), stromelysin (*MMP3*, *-11*, *-27*), and membrane-type (*MMP14*, *-16*, *-17*, *-24*, *-25*) subfamilies (27). Notably, collagenase expression remained unchanged, despite the increased collagen deposition observed during disease progression (19).

We investigated the FSHD animal model to study the dynamics, cellular sources, and functional roles of MMPs in FSHD pathology. Consistent with patient data, MMPs and related genes were strongly enriched in chronically DUX4-induced muscle, closely matching the expression profiles observed in FSHD STIR^+^ biopsies (39, 40). Longitudinal analysis revealed that several MMPs were upregulated as early as 1 day postinduction, indicating their involvement in the early stages of disease, similar to patterns seen in STIR^−^ muscle. Both the number and expression levels of MMPs progressively increased over time, highlighting a role in both acute and chronic phases of pathology. Comparative analysis of FSHD STIR^+^ muscle and DUX4-induced *iDUX4* mouse muscle revealed overlapping expression of key MMPs (*MMP2*, *-14*, *-17*, *-19*) and MMP-associated genes (e.g., *CYBA*, *FBLN1*, *TIMP1*), further supporting the relevance of the *iDUX4* model to human FSHD (13, 17, 59).

Our scRNA-Seq analysis identified FAPs and macrophages as the primary sources of *Mmp2*, *-14*, and *-19* in DUX4-affected muscle (Figure 3). While the function of MMP19 remains poorly understood, the roles of MMP2 and MMP14 in muscle regeneration and degeneration are well established (60–67). Our findings align with recent single-cell transcriptomic data from *mdx^5cv^* mice, which similarly identified FAPs and tenocytes as major contributors to *Mmp2* and *Mmp14* expression (60). Both MMP2 and MMP14 have been shown to promote adipogenesis in adipose tissue, skeletal muscle, and FAPs (61–64) and are implicated in the pathogenesis of several muscular dystrophies, including Duchenne, Emery-Dreifuss, and congenital muscular dystrophy 1D (65–67). MMP2, secreted by ECs and FAPs, degrades collagen IV, enabling SC migration from the basement membrane and facilitating the initiation of myogenesis (68–72). MMP14 contributes to fibrosis by cleaving intact collagen fibrils and activating latent TGFB1 released by Ly6C^+^ macrophages (73).

Beyond dystrophic conditions, MMP2 and MMP14 have been implicated in cardiac fibrosis and muscle remodeling associated with cancer cachexia (74). Macrophage-derived MMPs play key roles in ECM degradation, immune cell recruitment, and the regulation of inflammation during muscle regeneration (75–78). Notably, MMP14 mediates crosstalk between macrophages and muscle cells by regulating type I collagen turnover (79). Inhibition of MMP14 in dystrophic mouse models has been shown to reduce fibrosis, promote myofiber growth, and mitigate muscle damage (73).

Importantly, MMP14 expression is upregulated in response to IL-6, a pro-inflammatory cytokine elevated in FSHD and proposed as both a disease biomarker and therapeutic target (80–82).

To assess the functional impact of MMP inhibition on the DUX4-induced dystrophic phenotype, we treated *iDUX4* mice with batimastat. It is a small-molecule inhibitor that chelates the zinc ion within the active site of MMPs and was initially developed for clinical use in the treatment of malignant ascites (83, 84). We selected batimastat over other MMP inhibitors because of its pan-MMP activity and prior evidence of therapeutic benefit in *mdx* mice, where it reduced fibrosis and macrophage infiltration (33). Additionally, batimastat had been shown to inhibit adipogenesis in adipogenic progenitor cells, suppress FAP-mediated adipogenic differentiation, and decrease fat accumulation in dysferlinopathic muscle (64). Consistent with findings from other dystrophic models, batimastat treatment in *iDUX4* mice led to reduced ECM deposition, decreased infiltration of FAPs and macrophages, and improved myofiber size distribution in DUX4-affected muscles (Figures 4 and 5). These results provide strong evidence that MMPs actively contribute to DUX4-induced muscle pathology and support the therapeutic potential of pan-MMP inhibition in mitigating mild pathological features of FSHD.

Taken together, our study demonstrates that MMPs are actively involved in multiple stages of FSHD-associated muscle pathology, from early disease onset to the chronic phase. This highlights their potential as biomarkers for predicting disease onset, tracking progression, and evaluating treatment efficacy. More importantly, MMPs emerge as promising therapeutic targets for mitigating muscle degeneration and enhancing the regenerative niche in FSHD.

Our findings further validate the *iDUX4* mouse model as a robust platform for studying the molecular and cellular mechanisms driving FSHD progression. Single-cell resolution analyses revealed FAPs and macrophages as the primary sources of MMPs and key mediators of fibroadipogenic remodeling in DUX4-induced muscle damage. Importantly, we provide proof-of-principle evidence that pharmacological inhibition of MMPs can effectively ameliorate pathology in mildly affected muscle. Future studies should aim to dissect the specific roles of individual MMPs in the dystrophic process and to identify potent, selective MMP inhibitors with high efficacy and minimal off-target effects. In parallel, testing of a wider array of antifibrotic agents, alone or in combination with antiinflammatory treatments, should be prioritized as a complementary strategy for FSHD. Even with the emergence of direct anti-DUX4 therapies, targeting fibrosis will be essential for restoring the muscle’s regenerative potential and achieving meaningful functional recovery.

## Methods

### Sex as a biological variable.

Transcriptional analyses of patients with FSHD were conducted on both men and women, with no differences observed. In vivo experiments were performed exclusively on female mice because male mice of the *iDUX4pA* model are not viable due to FSHD-nonspecific pathology caused by the transgenes (13). Nevertheless, we do not anticipate significant differences between male and female mice.

### Experimental design.

The objective of this study was to determine the contribution of MMPs to the dystrophic process in FSHD. We conducted transcriptomic analyses using publicly available RNA-Seq datasets from 90 individuals, comprising both patients with FSHD and healthy controls. To further investigate the role of MMPs in vivo, we utilized the *iDUX4* FSHD mouse model to examine MMP involvement in DUX4-induced muscle degeneration. Sample sizes for animal studies were determined based on power calculations and prior experience with this model system. Predefined termination criteria were established and approved as part of the institutional animal care protocol. Experimental replicates and sample sizes are provided in the corresponding figure legends. No data were excluded from the analyses. Mice were randomly assigned to experimental groups, and all biological samples were coded to ensure blinded data collection and analysis.

### Mice.

All in vivo experiments were conducted at the University of Minnesota Research Animal Resources facility under a protocol approved by the IACUC (protocol number 2206-40184A). Four-week-old female mice were housed in a temperature- and humidity-controlled environment with a 12-hour light/12-hour dark cycle and provided with food and water ad libitum. For acute induction of DUX4, *iDUX4* mice developed before (13) were treated with i.p. injections of dox at 100 mg/kg, dissolved in PBS. For longer term induction (10 days and 16 weeks), mice were fed dox-containing chow (Envigo, 625 mg/kg), which corresponds to an approximate daily dose of 100 mg/kg, based on an average consumption of 4 g of food per day. Uninduced *iDUX4* mice or *WT* littermates treated with dox were used as controls. The specific control group used (*iDUX4* or *WT*), along with the number of mice per group, is indicated in each figure legend. For the batimastat study, both *WT* and *iDUX4* mice were fed dox-supplemented chow (62.5 mg/kg; Envigo) for 20 days (13, 16). On day 10, *iDUX4* mice were randomly divided into 2 groups. One group received batimastat (2 mg/kg, i.p.) every other day for the remaining 10 days, while the other group served as the disease control and received vehicle injections of equal volume. Body weight and muscle mass were recorded at the conclusion of the experiment.

### Muscle histology.

OCT-frozen 10 μm TA muscle sections were used for H&E staining to visualize myofibers and nuclei and for Sirius red/Fast Green staining to assess fibrosis (13). To quantify histological changes, the size and number of myofibers and the level of fibrosis were measured using ImageJ (NIH) and CellPose (https://www.cellpose.org). For immunofluorescence, tissue sections or sorted FAPs were fixed in 4% PFA for 10 minutes, permeabilized with 0.3% Triton X-100 for 30 minutes, and incubated overnight at 4°C with primary antibody against collagen VI (1:200, Proteintech), MMP2 (1:500, Proteintech), MMP14 (1:500, Proteintech), PDGFRα (1:200, BioLegend), F4/80 (1:200, BioLegend), and Ki-67 (1:400, BioLegend), followed by secondary antibody conjugate to Alexa Fluor 488, 555, or 647 (1:500, Thermo Fisher Scientific) for 2 hours at room temperature. Nuclei were visualized with DAPI (1:5,000, Sigma). Images were acquired using a microscope (Axio Observer Z1, ZEISS; and MICA Microhub, Leica).

### RNA isolation and RT-qPCR.

RNA was isolated from snap-frozen gastrocnemius using TRIzol (Invitrogen) and Direct-zol RNA Miniprep Kit according to the manufacturer’s instructions (Zymo Research). RNA concentration was determined by NanoDrop 2000 spectrophotometer (Thermo Fisher Scientific). A total of 1 μg RNA treated with DNase was reverse-transcribed into cDNA using oligo-dT primer and the cDNA synthesis kit (Applied Biosystems). RT-qPCR was performed using Premix Ex Taq Master Mix (Probe or SYBR Green, Takara) and commercially available TaqMan probes from Applied Biosystems (*Bmp4*: Mm00432087_m1; *Gapdh*: Mm99999915_g1; *Pdgfra*: Mm00440685_g1; *Wfdc3*: Mm01243777_m1), except for *DUX4*, which was detected using FAM-labeled probe (TCTCTGTGCCCTTG TTCTTCCGTGAA) or custom-designed primers (*Col1a1*: F, 5′ GAGCGGAGAGTACTGGATCG 3′ and R, 5′ TACTCGAACGGGAATCCATC 3′; *Col3a1*: F, 5′ TGGTCCTCAGGGTGTAAAGG 3′ and R, 5′ GTCCAGCATCACCTTTTGGT 3′; *Tgfb1*: F, 5′ CTCCCGTGGCTTCTAGTGC 3′ and R, 5′ GCCTTAGTTTGGACAGGATCTG 3′; *Mmp2*: F, 5′ CAAGTTCCCCGGCGATGTC 3′ and R, 5′ TTCTGGTCAAGGTCACCTGTC 3′; *Mmp17*: F, 5′ GGCAGTATGTTCCTGCACTTCA 3′ and R, 5′ GCTAGCAVTGCCCTCAGGAT 3′). Gene expression levels were normalized to that of *Gapdh* and analyzed using the ΔCT method.

### FACS analyses.

For single-cell suspension, quadriceps muscles were digested using collagenase type II and dispase, as previously described (16). Mononuclear cells were identified by flow cytometry using the following antibodies: PE-Cy7–conjugated anti-CD45 (clone 30-F11, BD Biosciences), APC-conjugated anti-CD31 (clone 390, eBioscience), PE-conjugated anti-PDGFRα (CD140A, clone APA5, BD Biosciences), PE-conjugated anti-SCA1 (clone D7, eBioscience), APC-conjugated anti-CD11b (clone M1/70, eBioscience), PE-conjugated anti-CD68 (clone FA-11, BioLegend), and PE-conjugated anti–GR-1 (clone RB6-8C5, eBioscience). Samples were run on a FACSAria II (BD Biosciences), and data were analyzed using FlowJo (BD Biosciences). Propidium iodide staining was used to discriminate live and dead cells during analysis.

### ATP luminescence assays.

CD45^–^CD31^–^PDGFRα^+^ FAPs were isolated from the quadriceps of *WT* and chronically induced *iDUX4* mice, then expanded in DMEM supplemented with 20% fetal bovine serum, 1× penicillin/streptomycin, and 2.5 ng/mL recombinant human basic FGF. At passage 3, cells were plated in 96-well plates at a density of 4 × 10³ cells per well in growth medium and cultured at 37°C under 5% CO_2_ and 5% O_2_. The following day, cells were treated with either 10 μM batimastat or 1 mM doxorubicin. After 24 hours, cell viability was assessed using the CellTiter-Glo Luminescent Cell Viability Assay Kit (Promega), following the manufacturer’s instructions. Luminescence, indicative of cell viability, was measured using the BioTek Cytation 3 (Agilent).

### Selection of MMP family and MMP-associated genes.

The MMP gene family consists of 23 members (35–37). To identify MMP-associated genes, we used the STRING database (version 12.0; https://string-db.org), focusing on protein-protein interactions with a confidence score above 0.4 to ensure reliable associations. The lists of human and mouse MMP and MMP-associated genes used in our analyses are presented in Supplemental Tables 1 and 2.

### Bulk RNA-Seq analysis.

The data for STIR^−^ and STIR^+^ in patients with FSHD were obtained from Banerji’s dataset (EGAS00001007350) in the European Genome-phenome Archive. This dataset contains paired-end RNA-Seq data from 48 muscle biopsy samples, including isogenic STIR^−^ and STIR^+^ biopsies from 24 patients with FSHD, and 11 control vastus lateralis muscle biopsies from unaffected individuals. In Wang’s dataset (GSE115650), muscle biopsies were collected from 34 patients with FSHD and 9 unaffected individuals at the initial visit. A total of 22 STIR^+^ and 12 STIR^−^ samples were sequenced using the Illumina HiSeq 2500 platform. Follow-up biopsies from the same cohort were analyzed in Wong’s dataset (GSE242912). Bilateral TA biopsies were performed for each participant, resulting in 64 RNA-Seq samples (23 STIR^+^, 41 STIR^−^, and 9 controls) that passed quality thresholds. RNA was extracted using TRIzol and sequenced with single-end 100 nt reads on the Illumina NovaSeq 6000 platform. In Tasca’s FSHD dataset (GSE26852), gene expression profiling was performed using the Illumina HumanHT-12_V3_0_R1 BeadChip. This dataset includes 15 muscle samples: 7 from healthy controls, 4 from STIR^−^ patients, and 4 from STIR^+^ patients. For DMD, data were obtained from Dorsey and Ward’s dataset (GSE38417), which includes 6 control and 16 DMD samples profiled using the Affymetrix Human U133 2.0 array. Only patients with DMD aged 3–8 years were included in the analysis. An additional DMD dataset by Pescatori (GSE6011) contains 22 DMD and 14 control muscle samples. All patients with DMD were diagnosed based on the absence of dystrophin immunoreactivity in quadriceps biopsies, and none had received corticosteroid treatment at the time of biopsy. Control samples were selected from diagnostic procedures in which no neuromuscular pathology was identified. For DM and BMD, data from Bachinski’s study (GSE13608) were used. This dataset includes 6 control samples, 10 DM1 and 20 DM2 biopsies (total 30 DM), and 5 BMD samples, profiled using the Affymetrix Human Genome U133 Plus 2.0 Array. For LGMD 2L, data were obtained from Claeys’ dataset (GSE202745). Muscle biopsies were taken from 16 male LGMD 2L patients and 15 age-matched male controls, covering 3 thigh muscles with different degrees of involvement: semimembranosus (severe), vastus lateralis (moderate), and rectus femoris (mild). A total of 41 LGMD 2L and 43 control RNA-Seq samples were generated using the Illumina NovaSeq 6000 platform.

Depending on the analysis context, batch effects were handled either by applying ComBat normalization across merged datasets or by performing within-dataset comparisons using matched case–control samples. Heatmaps of differentially expressed genes were generated using the pheatmap package. Volcano plots were constructed using ggplot2, with gene labels annotated via ggrepel.

For transcriptional analyses of DUX4-affected muscle, *iDUX4* mice were induced with a daily dose of 100 mg/kg dox by i.p. injection, while uninduced *iDUX4* mice served as controls. RNA was isolated from gastrocnemius muscle using the Direct-zol RNA Miniprep Kit. Library preparation and bulk RNA-Seq were performed at GENEWIZ, following previously described protocols (16). Briefly, strand-specific RNA-Seq libraries were prepared using the NEBNext Ultra II Directional RNA Library Prep Kit (New England Biolabs) following the manufacturer’s instructions. Enriched RNA was fragmented (8 minutes at 94°C), and first- and second-strand cDNA synthesis was performed, with dUTP incorporated during second-strand synthesis. After 3′ adenylation, indexed adapters were ligated, and libraries were enriched by limited-cycle PCR. Sequencing libraries were clustered on a flow cell and sequenced on an Illumina platform using a 2 × 150 bp paired-end configuration. Image analysis and base calling were performed with Illumina control software, and raw data were converted to FASTQ format and demultiplexed using bcl2fastq (v2.20) allowing 1 mismatch in index identification.

Differential expression analysis of bulk RNA-Seq data was conducted using the limma package (v3.58.1) in R (v4.4.0). Gene expression values were log2-transformed prior to differential expression analysis using the limma package. Genes with an adjusted *P* < 0.05 and absolute log2 fold change ≥ 1 were considered differentially expressed. GSEA was performed using the GSEA software (v4.3.2, Broad Institute). Gene lists were ranked by Signal2Noise, and custom gene sets corresponding to MMP family genes and MMP-associated genes (Supplemental Tables 1 and 2) were curated and used as the input database. Enrichment scores and significance values were calculated using default parameters (1,000 permutations, phenotype permutation type). Pathways with FDR < 0.25 were considered enriched, and leading-edge subsets were extracted for further visualization. Volcano plots were generated using the ggplot2 package (v3.4.4). Log2 fold changes were plotted against –log10 adjusted *P* values, and significance thresholds (adjusted *P* < 0.05 and |log2FC| > 1) were applied to distinguish significantly up- and downregulated genes. Selected genes of interest were highlighted and labeled manually for clarity. Heatmaps were constructed using the pheatmap package (v1.0.12). A curated list of biologically relevant genes, including MMP family members and MMP-associated genes, was used for visualization, regardless of statistical significance. Gene expression values were log2-transformed and truncated to a fixed range to ensure consistent color scaling. Where applicable, statistical significance (adjusted *P* < 0.05 and |log2FC| > 1) was annotated directly on the heatmap using asterisks.

### Samples and library preparation for scRNA-Seq.

scRNA-Seq was performed on 4-week-old *iDUX4* mice (*n* = 4) following 10 days of induction with dox chow (625 mg/kg). Age-matched, dox-treated *WT* mice (*n* = 5) served as controls. Single-cell suspensions were prepared from pooled skeletal muscles, 1 TA, 1 gastrocnemius, and 1 quadriceps muscle per mouse, using mechanical dissociation followed by collagenase/dispase digestion. Red blood cells were removed with 1× Red Blood Cell Lysis Buffer (Invitrogen), and dead or dying cells and debris were eliminated using the LeviCell EOS system (Levitas Bio) according to the manufacturer’s protocol. Live cells were stained with Acridine Orange/Propidium Iodide and counted using the LUNA-FL Cell Counter (Logos Biosystems). Equal numbers of viable cells from each mouse were pooled per group to obtain approximately 1 million cells. Cells were fixed using the Chromium Fixed RNA Kit (10x Genomics) and processed for library preparation with the Chromium Next GEM Single Cell Fixed RNA Sample Preparation Kit, targeting approximately 10,000 cells per sample. Libraries were sequenced on an Illumina NextSeq 2000 platform, generating approximately 10,000 reads per cell. Library preparation and sequencing were conducted at the University of Minnesota Genomics Center.

### Preprocessing scRNA-Seq data and cell type annotation.

Raw sequencing data were processed using Cell Ranger v9.0.1 (10x Genomics) with alignment to the mm10 mouse reference genome. Gene-barcode matrices were filtered to retain high-quality cells. Specifically, cells expressing fewer than 200 genes, or more than 10% mitochondrial transcripts, were excluded. Downstream analysis was conducted in R using the Seurat (v5.2.1) package (85). Data from control and *iDUX4* groups were normalized using SCTransform, and integrated using Seurat’s reciprocal principal component analysis–based integration workflow. The top 30 principal components were used for dimensionality reduction via UMAP. Cells were clustered using Seurat’s FindNeighbors and FindClusters functions. The optimal resolution parameter (0.2) was selected based on cluster stability visualized using the clustree (v0.4.4) package (86). Clusters were annotated manually based on canonical marker gene expression and further validated using the scMayoMap reference-based annotation tool (Supplemental Table 3) (87). Differential expression analysis was performed using the MAST method implemented in Seurat’s FindAllMarkers function, identifying genes with average log2 fold change and adjusted *P* values. Only positive markers with adjusted *P* < 0.05 were retained for heatmap visualization. Gene expression was visualized using UMAP plots, violin plots, bubble plots, and heatmaps. Unless otherwise specified, gene expression values were displayed as log-transformed normalized expression (log1p). In bubble plots, log2 fold changes and expression percentage differences between conditions (control vs. *iDUX4*) were used to reflect both magnitude and statistical significance. Violin plots were split by condition, and statistical comparisons were performed using 2-sided *t* tests with significance thresholds annotated directly on plots.

### Statistics.

GraphPad Prism software was used to analyze the data. Differences between groups were evaluated using the 2-tailed Student’s *t* test, multiple unpaired *t* tests, or 1-way ANOVA followed by Tukey’s post hoc tests. Differences were considered significant at *P* values of 0.05 or lower.

### Study approval.

Animals were maintained under protocol 2206-40184A, approved by the University of Minnesota IACUC.

### Data availability.

Sequencing reads and processed data have been deposited in the NCBI Gene Expression Omnibus under accession numbers PRJNA1300509 and PRJNA593958 (https://www.ncbi.nlm.nih.gov/sra?linkname=bioproject_sra_all&from_uid=1300509). Processed datasets and associated image files are available upon request. Supporting Data Values for all figures are provided in a single Excel file.

## Author contributions

Investigation was done by UJ, EW, HA, AM, KC, and DB. Analysis was done by UJ and DB. Bioinformatics was done by EW. Materials were provided by MK. Design and supervision were done by DB. Funding acquisition was done by DB. Manuscript preparation was done by UJ, EW, and DB. The order of the co–first authors was determined based on their relative contributions to the project, with UJ contributing primarily to the wet lab experiments and EW to the computational analyses.

## Funding support

This work is the result of NIH funding, in whole or in part, and is subject to the NIH Public Access Policy. Through acceptance of this federal funding, the NIH has been given a right to make the work publicly available in PubMed Central.

• Grant to DB from the NIH (R01 AR081228).

• Friends of FSH Research to DB.

• Regenerative Medicine Minnesota (RMM 072523 DS 004) to DB.

• University of Minnesota Genomics Center Pilot Sequencing Program to DB.

• NIH (R01 AR055685) to MK.

## Supplementary Material

Supplemental data

Supporting data values

## Version 1 09/18/2025

In-Press Preview

## Version 2 11/10/2025

Electronic publication
