# Supplementary material for: Matrix metalloproteinases are hallmark early biomarkers and therapeutic targets in FSHD
Source: JCI Insight. 2025 Sep 18;10(21):e195104. doi: 10.1172/jci.insight.195104 (PMC12643495; doi:10.1172/jci.insight.195104)
Supplement: Supplemental data [file jciinsight-10-195104-s018.pdf]

Supplemental Figure 1

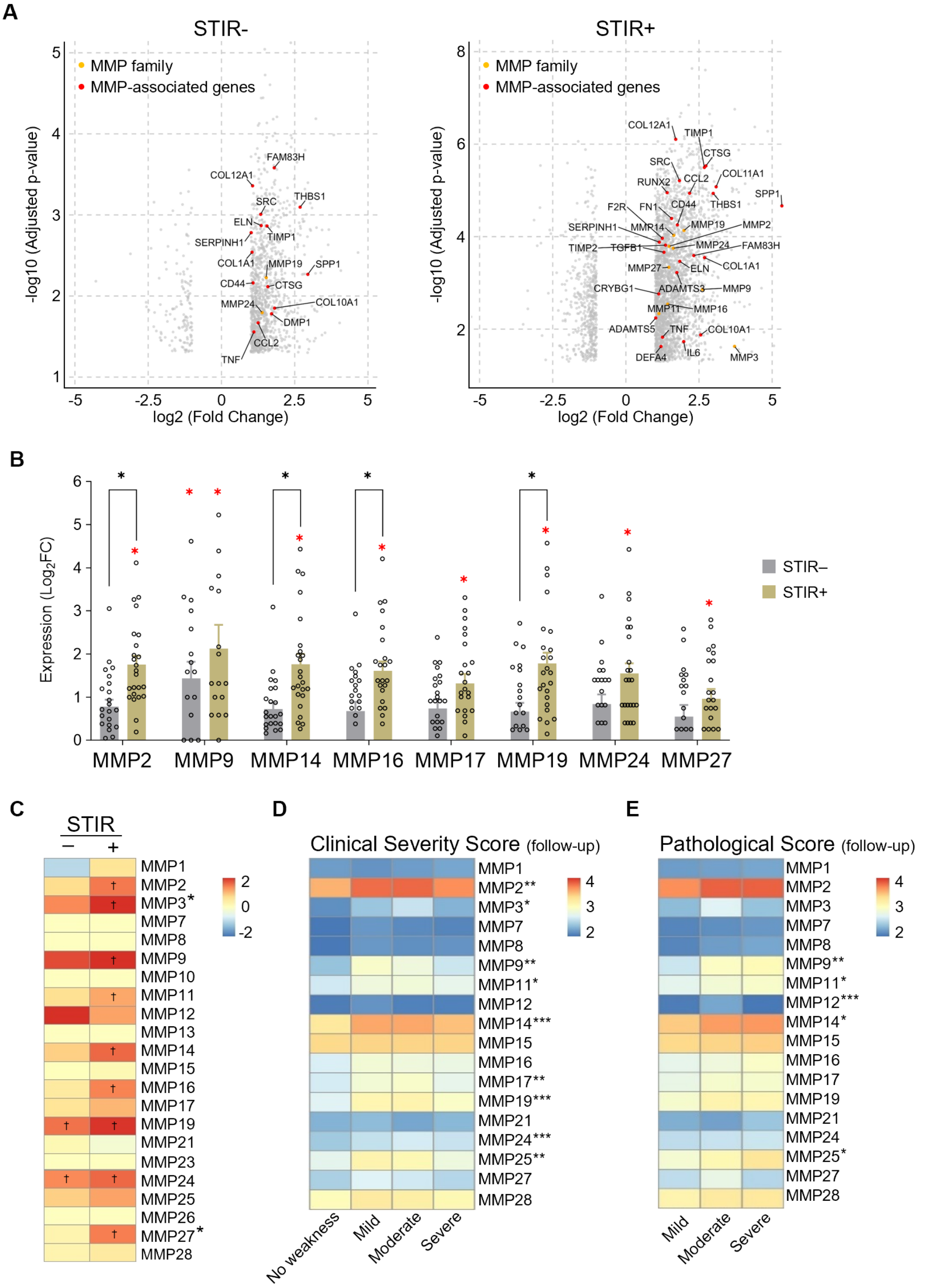

Supplemental Figure 1. MMPs expression in FSHD.

(A) Volcano plots displaying the differential expression of MMPs and MMP-associated genes in STIR− (left) and STIR+ (right) biopsies compared to healthy controls from Wang dataset.

(B) Bar graph showing MMP gene expression in FSHD biopsies from the Banerji study. Data are presented as mean ± SEM. Red asterisks indicate significant differences from the control healthy donors, while black asterisks denote differences between STIR− and STIR+ samples. $p < 0.05$ by multiple unpaired t-tests with FDR correction.

(C) Heatmaps illustrating MMP gene expression patterns in STIR− and STIR+ samples from the Wang dataset. Cross marks indicate statistical significance compared to control. †$p < 0.05$ by Student's t-test. Asterisks indicate significant differences between STIR− and STIR+. *$p < 0.05$ by Student's t-test.

(D-E) Heatmaps showing the correlation between clinical severity score (D) and pathological score (E) and MMP expression in follow-up visit biopsy samples from the Wang dataset. Asterisks indicate statistical significance. *$p < 0.05$, **$p < 0.01$, and ***$p < 0.001$ by one-way ANOVA.

Supplemental Figure 2

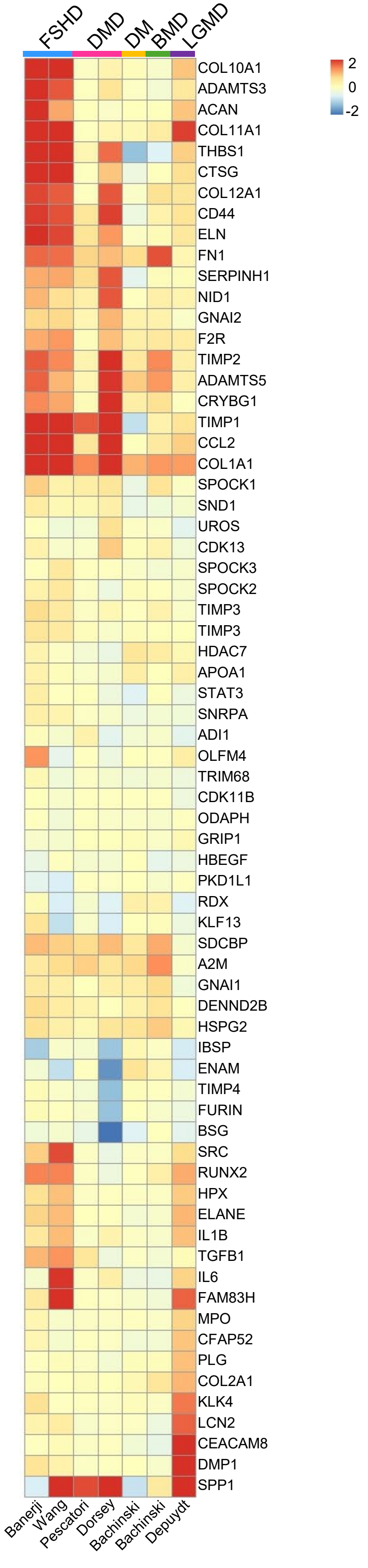

Supplemental Figure 2. Elevated MMP-associated gene expression in FSHD compared to other muscular dystrophies.
Heatmap depicting the differential expression of 79 MMP-associated genes in muscle samples from patients with FSHD, DMD, DM, BMD, and LGMD2L, compared to representative healthy control samples. Gene expression values are normalized and color-coded to indicate relative upregulation or downregulation across disease groups versus controls.

Supplementary Figure 3

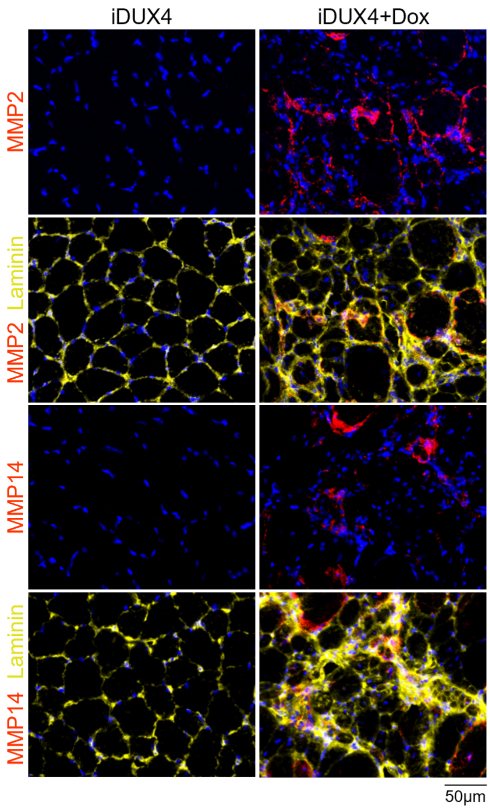

Supplementary Figure 3. MMP2 and MMP14 expression in DUX4-affected muscle.
Representative immunofluorescence images of quadriceps muscle from uninduced iDUX4 mice and iDUX4 mice induced for 16 weeks with 625 mg/kg doxycycline chow. MMP2 and MMP14 are shown in red, Laminin in yellow, and nuclei are counterstained with DAPI (blue). Scale bar: 50 μm.

Supplemental Figure 4

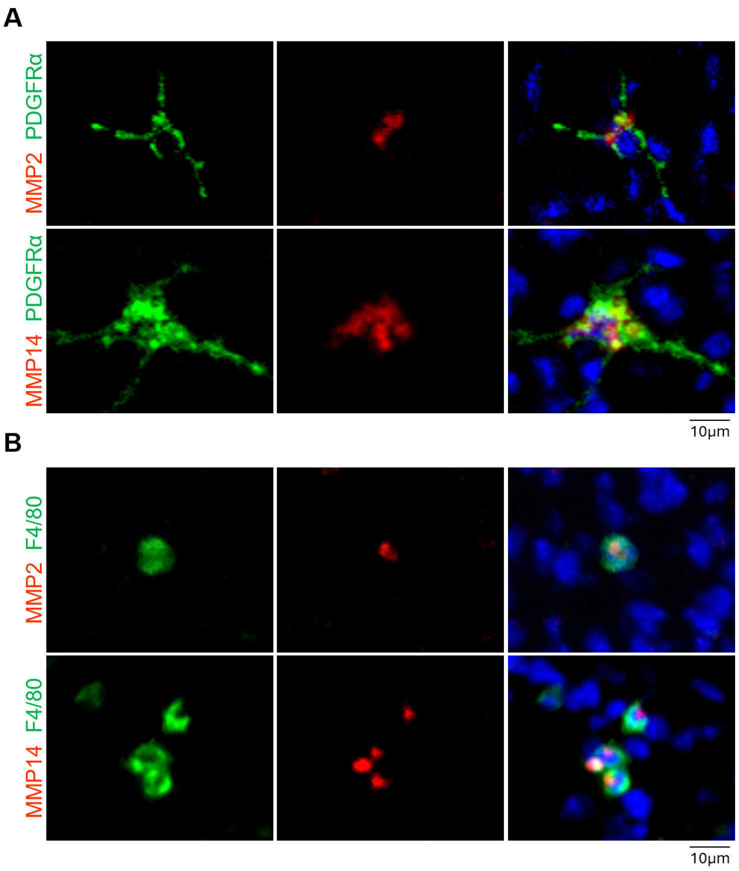

Supplemental Figure 4. MMP2 and MMP14 colocalization in FAPs and macrophages in iDUX4 mice.
(A) Representative immunofluorescence images of quadriceps muscle from a 4-week-old iDUX4 mouse induced for 10 days with 625 mg/kg doxycycline chow, showing MMP2 and MMP14 expression (both in red) in PDGFRA-positive (FAPs; green). Nuclei are counterstained with DAPI (blue).
(B) Representative images showing MMP2 and MMP14 (red) expression in F4/80-positive macrophages (green). Scale bar: 10 μm.

Supplemental Figure 5

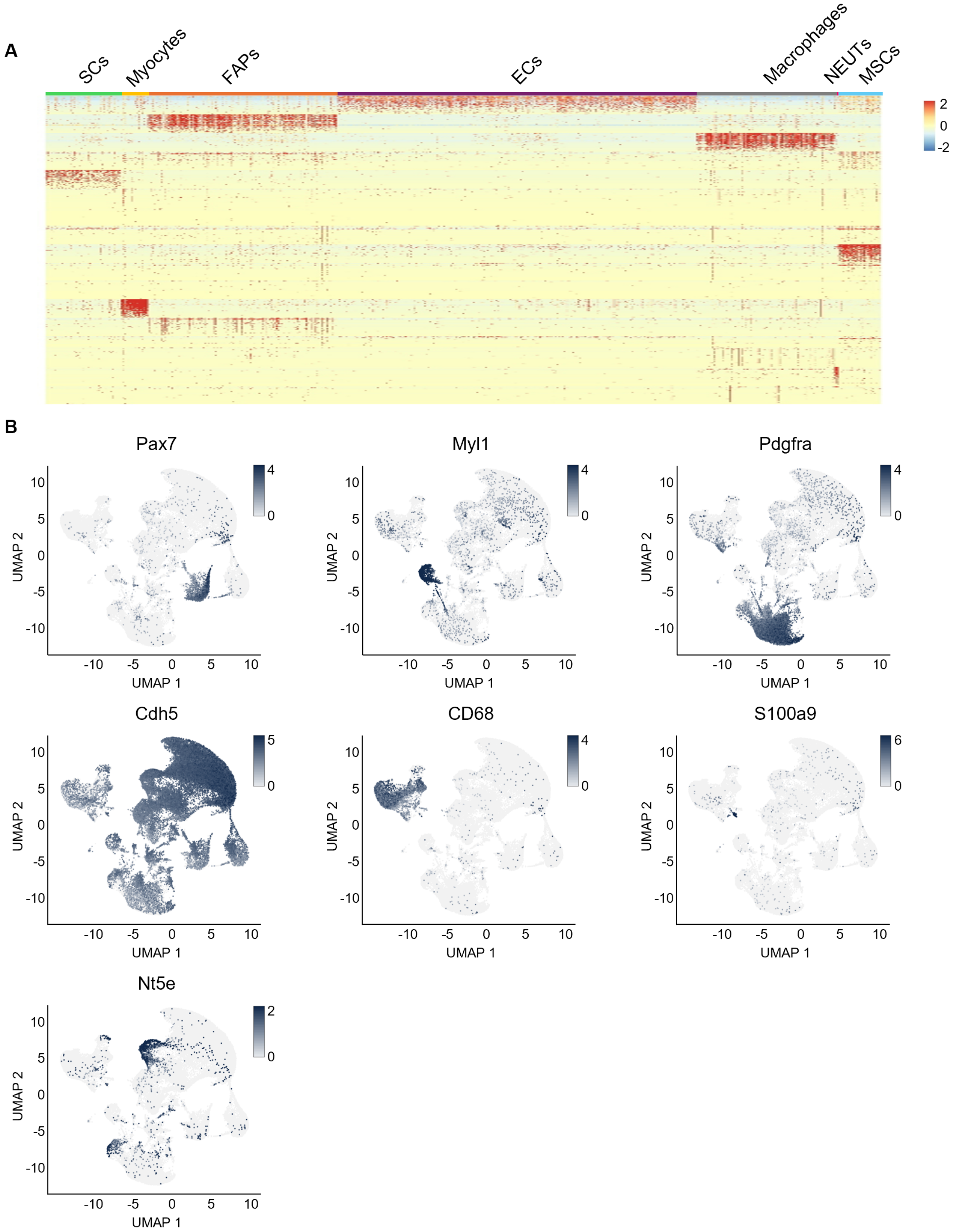

Supplemental Figure 5. Identification of cell types by single cell RNA-seq in iDUX4.
(A) Heatmap of genes defining 7 distinct cell types in control and 10-day post-DUX4 induced mice. Marker genes for cell type separation are listed in Supplementary Table 3.
(B) UMAP plot showing clear separation of cell populations based on their main markers: Pax7 for satellite cells, Myl1 for myocytes, Pdgfra for FAPs, Cdh5 for endothelial cells, CD68 for macrophages, S100a9 for neutrophils, and Nt5e for mesenchymal stem cells.

Supplemental Figure 6

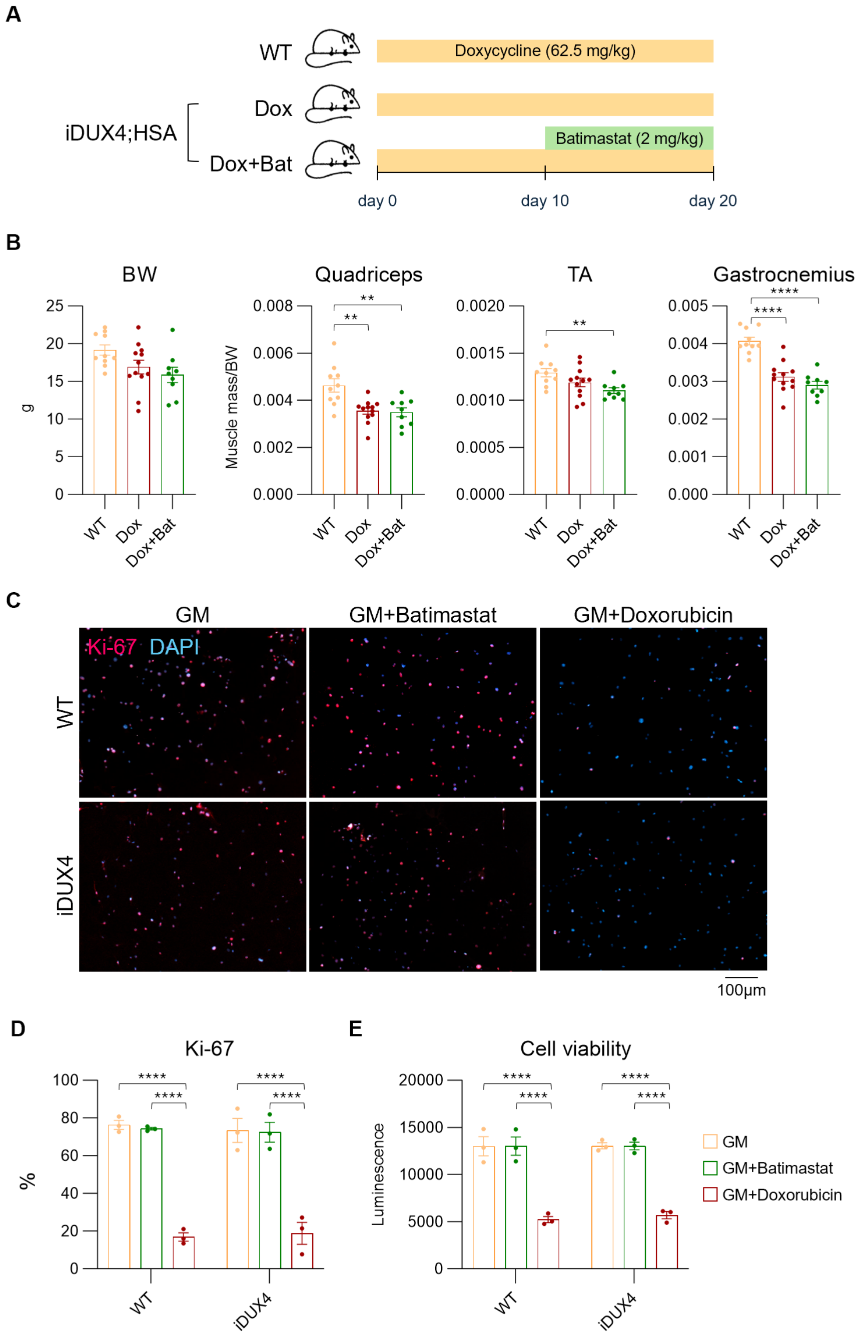

Supplemental Figure 6. Batimastat does not affect FAP viability.
(A) Schematic of the experimental timeline: iDUX4 mice were induced with doxycycline (62.5 mg/kg) for 20 days. Batimastat treatment (2 mg/kg) was administered via daily intraperitoneal injection starting on day 10. Experimental groups included wild-type littermate controls (WT, n=8), doxycycline-induced iDUX4 mice (Dox, n=9), and doxycycline-induced iDUX4 mice treated with Batimastat (Dox+Bat, n=8).
(B) Body weight and muscle mass (TA, quadriceps, and gastrocnemius+soleus) normalized to body weight at the experimental endpoint (day 20). Data are presented as mean ± SEM; *p < 0.05, **p < 0.01, ***p < 0.001, ****p < 0.0001 by one-way ANOVA.
(C) Representative immunofluorescence images of Ki-67 (red) and DAPI (blue) staining in FAPs isolated from WT and chronically induced iDUX4 mice after 24-hour treatment with 10 μM Batimastat or 1 mM doxorubicin. Untreated cells cultured in growth media served as controls.
(D) Quantification of Ki-67–positive cells normalized to total nuclei from the experiment shown in (C). Data are presented as mean ± SEM; ****p < 0.0001, by two-way ANOVA, n=3.
(E) ATP-based viability assay of FAPs treated for 24 hours with 10 μM Batimastat or 1 mM doxorubicin. Control cells (GM) were untreated and cultured in growth media. Data are presented as mean ± SEM; ****p < 0.0001, by two-way ANOVA, n=3.

Supplemental Table 1. List of human matrix metalloproteinases family and MMP-associated genes

Category	Gene list
Matrix metalloproteinase (MMP)	MMP1, MMP2, MMP3, MMP7, MMP8, MMP9, MMP10, MMP11, MMP12, MMP13, MMP14, MMP15, MMP16, MMP17, MMP19, MMP20, MMP21, MMP23, MMP24, MMP25, MMP26, MMP27, MMP28
MMP-associated genes	A2M, ACAN, ADAMTS3, ADAMTS5, ADI1, AMBN, AMELX, AMTN, APOA1, ATAMTS4, BSG, CCL2, CD44, CDK11A, CDK11B, CDK13, CEACAM8, CFAP52, COL10A1, COL11A1, COL12A1, COL1A1, COL2A1, CRYBG1, CTSG, DEFA4, DENND2B, DMP1, ELANE, ELN, ENAM, F2R, FAM83H, FN1, FURIN, GANI3, GNAI1, GNAI2, GRIP1, HBEGF, HDAC7, HPX, HSPG2, IBSP, IL1B, IL6, KLF13, KLK4, LCN2, MPO, NID1, ODAM, ODAPH, OLFM4, OR7G2, PKD1L1, PLG, RDX, RUNX2, SDCBP, SERPINH1, SND1, SNRPA, SPOCK1, SPOCK2, SPOCK3, SPP1, SRC, STAT3, TGFB1, THBS1, TIMP1, TIMP2, TIMP3, TIMP4, TNF, TRIM68, UROS, WDR72

* Kumar et al., (2022) Role of matrix metalloproteinases in musculoskeletal diseases.

* Jackson et al., (2010) Update of human and mouse matrix metalloproteinase families.

* Almeida et al., (2022) Matrix Metalloproteinases: From Molecular Mechanisms to Physiology, Pathophysiology, and Pharmacology.

* https://string-db.org (STRING version 12.0)

Supplemental Table 2. List of mouse matrix metalloproteinases family and MMP-associated genes

Category	Gene list
Matrix metalloproteinase (MMP)	Mmp1a, Mmp1b, Mmp2, Mmp3, Mmp7, Mmp8, Mmp9, Mmp10, Mmp11, Mmp12, Mmp13, Mmp14, Mmp15, Mmp16, Mmp17, Mmp19, Mmp20, Mmp21, Mmp23, Mmp24, Mmp25, Mmp27, Mmp28
MMP-associated genes	A2m, Acan, Adamts1, Adamts2, Adamts4, Adamts5, Ambn, Amelx, Amtn, Cd44, Cd68, Cfap52, Cfap53, Col12a1, Col2a1, Ctsk, Ctsg, Cyba, Cybb, Dcn, Edn1, Elane, Enam, Fam83h, Fn1, Furin, Gnai1, Gnai2, Gnai3, Hbegf, Heatr9, Hpx, Il1b, Il6, Klk1b4, Klk4, Lcn2, Lim2, Ltf, Mip, Mpo, Nid1, Odam, Pkd1l1, Plg, Sdc1, Slc35f1, Spp1, Src, Thbs1, Timp1, Timp2, Timp3, Timp4, Ubtf, Uros, Vegfa, Wdr72

* Kumar et al., (2022) Role of matrix metalloproteinases in musculoskeletal diseases.

* Jackson et al., (2010) Update of human and mouse matrix metalloproteinase families.

* Almeida et al., (2022) Matrix Metalloproteinases: From Molecular Mechanisms to Physiology, Pathophysiology, and Pharmacology.

* https://string-db.org (STRING version 12.0)

Supplemental Table 3. Specific marker genes and canonical cell type markers for clustering

Cluster	Cell types	Score	Gene list
1	ECs	0.30	KDR,EGFL7,CDH5,TSPAN13,LY6C1,FABP4,CD36,PECAM1,CAV1,GNG11,HSPB1,IGFBP7,CD34
2	FAPs	0.67	DPEP1,SCARA5,PDGFRA,LUM,PI16,C3,ADAMTS2,MFAP5,ENTPD2,DPT,FBLN1,MFAP4,HTRA3,SERPINF1,MMP2,DCN,GFPT2,LOXL1,ABCA8A,IGFBP6,GSN,BICC1,RNASE4,CCDC80,MEDAG,COL1A2,COL1A1,LSP1,MGST1,LPAR1,UGDH,CELF2,COL15A1,FBLN2,NAV1,CD34,S100A10,HEG1,S100A16
3	MΦs	0.42	CD86,MRC1(CD206),ADGRE1,CD83,FCGR1,C1QA,ITGAM,LAPTM5,ITGAX,C1QB,BCL2A1B,CCL3,IL1RN,CCL6,LYZ1,CTSS,CD14,FCER1G,CXCL2,TLR2,IL1B,GPNMB,LGALS3,CCL2,CD68,CSF2RB,S100A4,TNFAIP2,MPP1,SLCO2B1,ICAM1,H2AFZ,VIM,RHEB,CD36
4	SMCs	0.31	GJA4,RGS5,RASL12,NOTCH3,MRVI1,GJC1,AOC3,MYL9,ITGA1,MYLK,VASN,RRAD,PCP4L1,LMO7,MYH11,LAMB2,SMTN,HEXIM1,MSRB3,NRP2,DES,EHD2,FABP4,FAS,KIF1C,MUSTN1,TAGLN,ACTA2,BGN
5	Progenitor cells	0.14	MYF5,PAX7
6	Myocyte	0.21	MYF5,CDH15,PAX7,CHODL,FLNC,DES,NOTCH3,ITGA7,CXCR4,LAMA2,DMD
7	Myoblast	0.28	MYF5,FGFR4,CDH15,PAX7,MUSK,FLNC,DES,TNFRSF12A,ARL4D,EGFR,IGF2,STK40,CXCL1,ZBTB16
8	SCs	0.35	CALCR,MYF5,CDH15,PAX7,ASB5,NCAM1,PEG3,VCAM1,HEYL,RUNX1,SRXN1,TNFRSF12A,NOTCH3,ITGA7,CXCR4,SPRY1,DMD,CXCL1,CAV1
9	Schwann cells	0.99	CLDN19,BCAS1,MAG,MT3,MPZ,PRX,KCNA1,PLP1,S100B,SOX10,EGFL8,CNP,SEMA3B,GPR37L1,LDHB,PMP22,CADM4,CRYAB,ART3,ITGB4,STMN1,NDRG1,CD59A,CD82,EPHX1,TAGLN,LAMC1,ALDH2
10	SMCs	0.20	ACTA2,RGS5,TAGLN,FABP4,EHD2,HEXIM1
11	SMCs	0.58	CNN1,PLN,KCNAB1,KCNMB1,MAP3K7CL,LMOD1,SLC38A11,ITGA8,RBPMS2,MYH11,ACTA2,WFDC1,MRVI1,JPH2,TAGLN,MYL9,RASL12,PCP4L1,SPEG,SH3BGR,MYLK,AOC3,NEXN,MUSTN1,DMPK,RRAD,NOTCH3,SNCG,GJC1,GJA4,MSRB3,DES,OTUD1,SMTN,VASN,LAMB2,WWP2,PDE4DIP,SOD3,KIF1C,HEXIM1,BGN,FABP4
12	MPCs	0.29	NT5E,ENG,MCAM
13	MSCs	0.30	NT5E,ENG,CD34,LY6A,MCAM,ITGB1
14	SMCs	0.53	MAP3K7CL,SLC38A11,KCNAB1,PLN,HHIP,KCNMB1,LMOD1,WFDC1,RBPMS2,MRVI1,RASL12,GJA4,ITGA8,FBLN5,PCP4L1,MYH11,CNN1,RRAD,JPH2,SPEG,AOC3,ACTA2,SNCG,MYL9,GJC1,NOTCH3,TAGLN,NEXN,MYLK,MUSTN1,MSRB3,PDGFD,OTUD1,DMPK,ACKR3,RGS5,DES,WWP2,LMO7,SSPN,ITGA9,LAMB2,VASN,FAS,KIF1C,LOX,KAT2B,ITGA1,SEC24D,AKT2,EHD2,SOD3,HEXIM1,FABP4,PDE4DIP,BGN
15	Schwann cell	0.90	BCHE,NRN1,RXRG,LGI4,ASPA,GPR37L1,CADM4,CMTM5,SOX10,MATN2,KCNA1,PLP1,SCN7A,S100B,ITGB4,EGFL8,CNP,SEMA3B,FXYD1,BCAS1,ART3,CAB39L,CLDN19,CD59A,MAG,LDHB,APOD,CRYAB,MPZ,PRX,PMP22,STMN1,NDRG1,IFI27,LAMC1,MDH1,NF2,BAG3,PRDX5,NDUFV3,NDUFA1,CDKN1C,PLTP
16	Myocyte	0.87	MYL1,MYOZ1,MYH4,ACTN3,CKM,ACTN2,TNNI2,MYLPF,TMOD4,NEB,ACTC1,TNNC2,TNNT3,TTN,ACTA1,TCAP,MYH1,LDB3,ENO3,PYGM,DES,TPM2,FLNC
17	SMCs	0.16	LOX,SPON2,NOX4,OGN,ITGA8,BGN,VASN,SEC24D,FBLN5,MFAP5,LAMB2,MSRB3,AKT2,SOD3,OTUD1,NF2,MYLK,SMTN,ITGA1,EHD2,FABP4

18	Myocyte	0.27	THBS4,CHODL,MMP2,LAMA2,SULF1,WIPF1,NID1,IQSEC1,FLNC,IL4RA,TPM2
19	FAPs	0.33	MFAP4,FBLN1,SERPINF1,MMP2,LPAR1,COL1A1,COL1A2,IGFBP6,BICC1,DCN,ADAMTS2,CCDC80,RNASE4,LOXL1,GFPT2,LSP1,MFAP5,UGDH,LUM,GSN,ABCA8A,HEG1,COL15A1,NAV1,CD34,LY6A
20	Myocyte	0.24	EDN1,ITGA3,SULF1,DLL1,PTK2,ARHGAP26,DOCK1,IQSEC1,MEF2D,PXN,NID1
21	SMCs	0.26	FBLN5,GJA4,PDGFD,MSRB3,LMO7,ACKR3,LAMB2,FAS,SNCG,EHD2,WWP2,ITGA1,LOX,FABP4,KIF1C,MYLK,BGN,SMTN
22	MΦs	0.97	TPX2,BIRC5,CDK1,ANLN,UBE2C,NUSAP1,TYMS,STMN1,KPNA2,ITGAM,S100A4,IL1B,LGALS3,H2AFZ,LAPTM5,CTSS,CD83,KLHDC4,TLR2,ADGRE1,FCER1G,MRC1(CD206),CD14,CXCL2,C1QB,CSF2RB,C1QA,MPP1,CD68,VIM,NR2F6,TNFAIP2,RHEB,ICAM1,TRIM25,CD36
23	NEUT	0.95	S100A9,RETNLG,S100A8,CXCR2,TREM1,TREM3,MMP9,ASPRV1,IL1R2,ARG2,HDC,SELL,CSF3R,CLEC4D,HP,CCR1,CLEC4E,LCN2,JAML,IL1B,SORL1,NLRP3,CD33,C5AR1,ADAM8,SNX20,PTGS2,CCL3,ITGAM,CCL6,CXCL2,PSTPIP1,CD14,NCF1,TLR2,CCRL2,LRG1,LYST,ANXA1
24	NEUT	0.44	HP,LCN2,IL1R2,LRG1,SLC1A5,ADPGK,PECAM1
25	MΦs	0.54	WFDC21,APOC1,GPD1,ACP5,CCL2,G0S2,MPP1,GDA

ECs, endothelial cells; FAPs, fibroadipogenic progenitors; MΦs, macrophages; SMCs, smooth muscle cells; SCs, satellite cells; NEUT, neutrophils; MPCs, mesenchymal progenitor cells; MSCs, mesenchymal stem cells;
